# Test of Motor Proficiency Second Edition (BOT-2): Compatibility of the Complete and Short Form and Its Usefulness for Middle-Age School Children

**DOI:** 10.3389/fped.2019.00153

**Published:** 2019-04-18

**Authors:** Jan Jírovec, Martin Musálek, Filip Mess

**Affiliations:** ^1^Faculty of Physical Education and Sport, Charles University, Prague, Czechia; ^2^Department of Sport and Health Sciences, Technical University of Munich, Munich, Germany

**Keywords:** BOT-2, complete form (CF), short form (SF), psychomotoric, standard score, sensitivity, specificity, middle-age school children

## Abstract

**Background:** The Bruininks-Oseretsky Test of Motor Proficiency Second Edition (BOT-2) assesses the psychomotor development. It is available in two forms. According to several studies the BOT-2 short form (SF) provides significantly higher results than the BOT-2 complete form (CF). This might be due to the use of an inadequate type of scores when comparing results of the SF and the CF.

**Objective:** To verify whether the degree of psychomotor development assessed by the BOT-2 SF is comparable to the results of the BOT-2 CF in middle-age school children when using standard scores considering age and sex.

**Methods:** The research sample consisted of *n* = 153 neurotypical children (*n* = 69 girls, *n* = 84 boys) from 8 to 11 years (9.53 ± 0.85). The degree of psychomotor development was determined by the standard scores of the BOT-2 CF and BOT-2 SF—both considering sex and age. The conformity in results between the CF and the SF, the sensitivity and specificity of the BOT-2 SF and the relations between the results of each sub-test within the BOT-2 CF and the BOT-2 SF were analyzed.

**Results:** The BOT-2 SF provided a statistically significantly lower standard score *x* = 45.87 (±5.41) compared to the BOT-2 CF *x* = 47.57 (±8.29) *p* < 0.05 with middle effect size value, Hays ω^2^ = 0.09. The ROC analysis showed that the BOT-2 SF obtains sufficient sensitivity (84%) but poor specificity (42.9%) and AUC = 0.484 CI95% (0.31–0.62). Moreover, only 57% of total variance of the BOT-2 CF is explained by the relation between the results of the CF and the SF.

**Conclusion:** The BOT-2 SF does not provide practically significant different results compared to the BOT-2 CF when using a proper scale for comparing both versions. In addition, poor specificity of the BOT-SF suggests that the BOT-2 SF might be a useful tool to reveal mainly psychomotorically delayed but not above average (psychomotorically advanced) children. Further, due to the weak portion of a shared common factor, it remains still unclear whether the BOT-2 CF and the BOT-2 SF measure the same behavioral domain.

## Introduction

The human development is defined as changes influenced by interactions between genetic disposition and environmental factors. The psychomotor development, generally known as psychomotorics (1), is a complex process specific to each individual. Changes, both in physical and psychological development, are closely related to changes in motor functions (e.g., motor control, motor activity) (2–4). Within psychomotorics we encounter many concepts: e.g., sociomotor or sensory development, emotional or social development, and fundamental motor skills (FMS) (e.g., fine motor, gross motor, sensomotor, neuromotor) (5, 6). Previous research showed that an incomplete level of FMS in children later might cause problems in their psychological and social development (7–9). Therefore, the evaluation of psychomotorics already in childhood can help reveal deficits in motor development and targeted interventions can be carried out. In the evaluation of psychomotorics, a wide range of test batteries is used. Some of the most common test batteries are the TGDM-2 (10), the MABC-2 (11) or the BOT-2 (12).

The BOT-2 is a commonly used diagnostic instrument in the evaluation of the development of psychomotorics in the age range from 4 to 21. The BOT-2 is mostly used in the field of medicine focusing on children—e.g., by pediatricians, physiotherapists, physical education teachers in adaptive teaching (13, 14). The BOT-2 exists in two forms: the complete form (CF) and the short form (SF). The test allows to determine the level of FMS in the general population as well as in specific groups of children with mental disorders (15, 16). Four areas of psychomotorics are evaluated: (1) fine manual control—preciseness and integrity, (2) manual coordination—manual skill and coordination in the upper limbs, (3) physical coordination—bilateral coordination and balance.

The advantage of the BOT-2 CF and SF tests are their high reliability, rel = 0.9 to 0.97 (12, 17), Luca et al. (18) applying the BOT-2 SF to Australian Aboriginal children that were prenatally exposed to alcohol found that the BOT-2 SF obtains excellent reliability and is a suitable screening tool for determining motor deficits in this population.

Many studies (13, 15, 19–21) used the BOT-2 SF because the SF is less time-consuming (15–20 min per person) compared to the CF (45–60 min per person). Moreover, according to Bruininks and Bruininks—the authors of the BOT-2, (12), a strong correlation between the SF and CF existed *r* = 0.80 to 0.87. Further, the individual sub-tests of the BOT-2 as well as the overall standard score are standardized according to sex and age. It is also necessary to emphasize that, according to the authors of the BOT-2 (12), the BOT-2 SF is a screening tool whose results should determine whether any further assessment is necessary.

Although Bruininks and Bruininks (12) found acceptable the suggested four-factor model, which they verified using confirmatory factor analysis, several subsequent studies though pointed out possible psychomotor problems with this diagnostic tool especially with the BOT-2 SF. Using the Rasch Measurement Model, Brown (22) revealed that although all 14 items of the BOT-2 SF formed a unidimensional structure, nine items did not comply with the fit requirements, which means that the results of the 14 items “*cannot be summed together to calculate a composite score with confidence”* [(22), p. 100]. A more detailed view of the relations between individual sub-test items and the relevant overall sub-test score in the CF in middle-age school children (6–10 years) was provided in studies (23, 24). Brahler et al. (23) who investigated the aforementioned relations in four sub-tests (fine motor integration, fine motor precision, balance, strength) of the total of eight sub-tests, found a wide range of correlations in individual sub-test items in the CF with the relevant overall sub-test scores ranging from *r* = 0.07 to 0.86. In this study the items which are also in the BOT-2 SF, Copying Star and Copying Square showed low levels of correlation with the overall score of the Fine Motor Integration sub-test *r* = 0.232 and 0.264, respectively. Moreover, for other two items, Drawing Lines through Paths (crooked) and Walking Forward on a Line, an absolute ceiling effect was discovered, i.e., no dispersion of values [see more in Brahler et al. (23)]. Carmosino et al. (24), who continued in the design of the previous study (23) and investigated the relations between the overall sub-test score in the CF in the rest of the four sub-tests (manual dexterity, bilateral coordination, running speed & agility, upper limb coordination), revealed that the weakest correlation was generally identified between the bilateral coordination overall sub-test score and its items. In contrast to Brahler et al. (23) who stated that the investigated items in the four sub-tests, which are also included in the BOT-2 SF, obtain low correlations with the relevant overall sub-test score, Carmosino et al. (24) concluded that except for bilateral coordination, items from the other three sub-tests (manual dexterity, running speed and agility, upper limb coordination) sufficiently correlate with the overall sub-test score. However, a high level of correlation between items and sub-test scores in the CF and SF do not necessarily imply that the two versions provide comparable or even identical results for psychomotor development. Therefore, surprisingly, less attention has been given to the differences in the overall scores of the CF and the SF.

Venetsanou et al. (19), who compared the total score of the BOT-2 SF and the standard score of the BOT-2 CF in pre-school children (age range 4.5–5.5), revealed that the SF total point score (*M* = 58.72) significantly overestimates the overall result in comparison with the CF standard score (*M* = 47.38). Nevertheless, comparing the standard score of the CF and the total point score of the SF does not make sense since the SF total point score does not consider age and sex. According to the authors of the BOT-2, when comparing the SF's and CF's results, both the SF standard score, which considers participants' age and sex and the CF standard score must be used (12).

Although previous studies revealed possible psychometrics lacks of the BOT-2 SF (19, 22–24), there has been only limited information about the compatibility of results between the BOT-2 CF and BOT-2 SF as well as the sensitivity and specificity of the BOT-2 SF in children.

Therefore, the aim of this study is to verify the compatibility of the BOT-2 SF and BOT-2 CF in middle-age school children (8–11 years), using the standard score, which considers age and sex. Further, we will verify whether the BOT-2 SF obtains sufficient sensitivity and specificity to adequately identify the different degrees of psychomotor development.

## Methods

### Sample

The sample consisted of 153 middle-age school children aged 8 to 11 (*M* = 9.53 ± 0.85 years), (boys *n* = 84, girls *n* = 69). The sample was selected from an elementary school without any specific specialization and did not include any children who were mentally or neurological impaired. The testing took place in October 2016.

The procedures involved in our study were in accordance with the ethical standards of the responsible Czech national committee on human experimentation and with the Helsinki Declaration of 1975, as revised in 2000. The research was approved by the Ethics Committee of the Faculty of Physical Education and Sport, Charles University, and the parents of all participants signed an informed consent. The data were anonymized.

No differences regarding sex were found in basic characteristics: age, weight, height, and BMI (Table 1).

**Table 1:** Physical aspects of participants.

	**Total**		**Boys**		**Girls**	
	**Mean**	**SD**	**Mean**	**SD**	**Mean**	**SD**
Age	9.53	±0.85	9.60	±0.86	9.44	±0.83
Weight	35.84	±10.66	37.07	±11.78	34.34	±8.96
Height	139.66	±8.67	140.39	±8.87	138.78	±8.40
BMI	18.10	±3.72	18.48	±4.06	17.64	±3.23

*SD, standard deviation*.

### Measurements

The level of psychomotor development was assessed by the BOT-2 testing battery (12). The children were tested using the complete version of the battery (CF).

The BOT-2 CF contains 53 items divided into 8 sub-tests (concepts). Each sub-test includes six to seven tasks. Sub-tests are: fine manual control (1) fine motor precision and (2) integration; manual coordination (3) manual dexterity, and (4) upper-limb coordination; body coordination (5) bilateral coordination and (6) balance; strength and agility (7) speed and (8) strength [complete list of items in Bruininks and Bruininks (12)]. The measurement time of the BOT-2 CF for each participant ranges from 45 to 60 min.

The BOT-2 SF contains 14 items. For each sub-test one to two tasks are selected from the CF form. The results for the SF were obtained from the results of the CF.

In order to adequately compare the results of the SF and CF, we used the standard score for the CF and the standard score for the SF. Both scores represent normalized values, which consider age and sex of the participants (12).

In the BOT-2 CF and BOT-2 SF, participants receive a raw score, which is transformed to a point score. This point score is further transformed to a standard score, which considers age and sex (12).

### Data Collection

The data were collected in accordance with the BOT-2 manual (12) in the environment of the selected schools. The measurement was carried out in the morning (8 a.m. to 1 p.m.) instead of physical education classes. It was supervised by the examiner and one teacher. Each participant was tested individually at eight different stations in one room. One professionally trained examiner measured all participants. All data were recorded in given forms for the CF and SF and then copied into the ASSIST program.

### Statistical Methods

The Shapiro-Wilk test, the Martinez-Iglewicz test and the Kolmogorov-Smirnov test did not reject the normality of the data. The Two-Way Analysis of Variance (ANOVA) was used to establish the difference between the standard scores of the CF and SF with respect to gender. Pearson's product moment correlations between the CF and SF, as well as between the individual sub-tests and the standard scores in the CF and SF were calculated. The criteria for the statistical significance p < 0.05 and effect size (Hays ω^2^) were based on the recommended guidelines. The effect size magnitude was interpreted as follows: Hays ω^2^ (0.01–0.069) small influence effect, Hays ω^2^ (0.071–0.137) medium influence effect, Hays ω^2^ > 0.138 large influence effect. Pearson's product moment correlations between the CF and SF, as well as between the individual sub-tests and the standard scores in the CF and SF along with correlation ES Cohen q, were calculated. The correlation ES magnitude was interpreted as follows: small effect, Cohen q=0.1-0.3; medium effect, Cohen q = 0.3-0.5; large effect, Cohen q>0.5 (25, 26). For sensitivity and specificity of the BOT-2 SF the Receiver Operating Characteristic (ROC) analysis was used (27). The data were evaluated by the NCSS2007 program (Version 2007; NCSS, Kaysville, UT, USA).

## Results

The BOT-2 diagnostic tool was used to evaluate four areas of psychomotor development: fine manual control (fine motor precision and integration), manual coordination (manual dexterity and upper-limb coordination), body coordination (bilateral coordination and balance), strength and agility (running speed and agility, strength).

The results of Two-Way ANOVA showed a significant difference in the standard scores between the BOT-2 CF *x* = 47.57 (±8.29) and the standard score BOT-2 SF *x* = 45.87 (±5.41) *F*_(1, 151)_ = 15.34, *p* < 0.01 with medium effect size (ES) Hays ω^2^ = 0.09. The main effect of gender was not proved even though girls achieved greater results in both the BOT-2 CF and the BOT-2 SF *F*_(1, 151)_ = 3.24, *p* = 0.074, Hays ω^2^ = 0.02 (Table 2). The correlation between the CF and SF was *r* = 0.76.

**Table 2:** Results of the BOT-2 CF and BOT-2 SF, boys and girls.

**Sub-tests**	**Boys**	**Girls**
	**Mean ± SD**	**Mean ± SD**
Fine manual control	50.19 ± 9.80	51.44 ± 9.43
Fine motor precision	13.09 ± 4.21	13.59 ± 3.86
Fine motor integration	16.61 ± 4.58	17.97 ± 4.71
Manual coordination	49.66 ± 9.73	52.47 ± 8.96
Manual dexterity	15.01 ± 4.13	15.72 ± 4.37
Upper-limb coordination	15.02 ± 4.99	16.55 ± 4.75
Body coordination	42.90 ± 6.98	43.46 ± 8.08
Bilateral coordination	12.76 ± 3.87	12.98 ± 4.26
Balance	12.17 ± 3.27	12.00 ± 3.77
Strength and agility	48.38 ± 5.66	49.88 ± 6.01
Running speed and agility	11.63 ± 2.71	13.31 ± 2.79
Strength	16.88 ± 3.45	16.75 ± 3.29
BOT-2 CF	46.57 ± 7.66	48.79 ± 8.90
BOT-2 SF	45.19 ± 5.17	46.71 ± 5.60

*SD, standard deviation*.

When considering boys and girls together, the greatest differences in correlations between the sub-tests and the standard score of the CF and SF were found in fine manual control and body coordination. The lowest correlation in the CF was revealed between the CF standard score and the strength and agility sub-test (*r* = 0.65). On the other hand, these correlations were the most stable regarding the CF and SF. A separate correlation analysis of each sex revealed the greatest difference of correlation between the body coordination sub-test and the standard score of the CF and the standard score of the SF in girls (Table 3).

**Table 3:** Pearson correlation coefficient of the BOT-2 CF and BOT-2 SF–total, boys, girls.

**Sub-test**	**BOT-2 CF standard score (boys and girls)**	**BOT-2 SF standard score (boys and girls)**	**Cohen *q***
Fine manual control	0.78	0.57	0.39^**^
Manual coordination	0.79	0.68	0.24^*^
Body coordination	0.72	0.45	0.42^**^
Strength and agility	0.65	0.60	0.08
	**BOT-2 CF standard score girls**	**BOT-2 SF standard score girls**	
Fine manual control	0.82	0.61	0.44^**^
Manual coordination	0.77	0.65	0.24^*^
Body coordination	0.79	0.48	0.59^***^
Strength and agility	0.72	0.60	0.21^*^
	**BOT-2 CF standard score boys**	**BOT-2 SF standard score boys**	
Fine manual control	0.76	0.54	0.39^**^
Manual coordination	0.81	0.70	0.26^*^
Body coordination	0.65	0.43	0.31^**^
Strength and agility	0.57	0.58	0.01

significance of correlation differences between CF and SF:

*** large ES, Cohen q>0.5;

** medium ES, Cohen q = 0.3-0.5;

* *small ES, Cohen q = 0.1-0.3*.

In the next step we analyzed the sensitivity and specificity of the BOT-2 SF. In the ROC analysis procedure we worked with a 5-point Likert scale established according to Bruininks and Bruininks (12) manual. In both the BOT-2 CF and SF this scale is used to transform the standard score to final categories (1 - well below average; 2 - below average; 3 - average; 4 - above average; 5 - well above average) (12). The ROC analysis assessing the sensitivity and specificity found that the BOT-2 SF obtains high sensitivity (84%) but poor specificity (42.9%) with 76.5% accuracy and poor value of *Empirical Area Under Curve Analysis* (AUC) = 0.484 CI95% (0.31–0.62) in comparison to BOT-2 CF. The ROC analyses conducted for boys and girls separately showed that high sensitivity of the BOT-2 SF (boys = 82.6%, girls = 85.7%) along with its low specificity (boys = 53%, girls = 30.7%) is stable regardless of gender.

## Discussion

The aim of this study was to verify whether the degree of psychomotor development assessed by the BOT-2 SF is similar compared to the results of the BOT-2 CF in middle-age school children. Further, the degree of relations between each sub-test and the two test forms (SF and CF) was assessed.

We used the standard score for the CF and the standard score for the SF, which both consider age and sex, when comparing the SF's and CF's results (12).

The results showed that the BOT-2 SF underestimates the degree of FMS compared to the BOT-2 CF. Although the differences between the CF and SF were statistically significant, the effect size (ES) achieved only medium level Hays ω^2^ = 0.09. The main effect of gender was not proved even though girls achieved greater results in both the BOT-2 CF and BOT-2 SF.

According to literature (12, 13, 16), the more comprehensive CF assesses the current degree of psychomotor development better and in more detail compared to the SF. But this does not mean that the BOT-2 CF necessarily provides higher scores. When we look closely at each sub-test of the CF, we see that participants have to pass several items within each sub-test. This means that the participants have a possibility to encounter more motor experience during the tests and have more time to adapt to it. This approach is called local independence. According to methodologists (28, 29), the assumption of local independence is typically violated in item sets and can lead to many problems including overestimating the degree of the measured trait or misleading specificity of the determined constructs (30). These specific implications were thoroughly studied by the Item Response Theory (IRT) approaches (31–33).

In our opinion, the variance of the amount of items in each sub-test of the CF and SF might cause the violation of local independence, which can further influence the final difference in the observed degree of psychomotor development. Although the results of Brown's (22) study, in which the IRT method was used, in particular the Rasch Measurement Model, did not confirm the violation of local independence in the BOT-2 short form, we assume that the similarity in the content of some motor tests (e.g., copying star, copying square) might result in the violation of local independence, especially in the assessment of neurotypically developed children.

Our findings are not in line with previous studies (19, 34), which compared the results of the SF and CF. These studies compared the CF standard scores and the SF total point scores However, the SF total point score does not consider age and sex specifics. The authors of the BOT-2 emphasize, when comparing the SF and CF results, that the SF standard score and the CF standard score, which consider factors of age and sex of the participants, must both be used (12). Venetsanou et al. (19) compared the SF total point score *M* = 58.72 (±7.28) and the CF standard score *M* = 47.38 (±9.43) in 144 children (74 males, 70 females; mean age 5.2). They found out that the SF significantly overestimates the standard score compared to the CF. Holický (34) came to the same conclusion: SF *M* = 75.95 (±2.95); CF *M* = 58.9 (±5.99). If we had considered the total point scores, our results would have been in line with the previous findings: SF total point score *M* = 64.87 (±4.57) and CF standard score *M* = 47.57 (±8.29).

The correlation between the standard scores of the CF and SF was *r* = 0.76. This finding is in line with previous research (12, 19, 35). In these studies, correlations between the SF and CF forms were *r* = 0.80 and higher (12) *r* = 0.8; (19) *r* = 0.85), which, according to the authors, indicated that each form ranks children's performance in a similar way. Kambas et al. (35) claimed that the BOT-2 SF is valid enough to estimate the motor proficiency of boys and girls within the same age range as the complete form of the battery. They found that the BOT-2 SF is a valid age appropriate screening tool to test the motor proficiency of normal preschool and primary school children in Greece. However, when we consider the validation or test equivalence of a method, we should also calculate the determination coefficient *r*^2^, which shows how much variance in a dependent variable is explained by the relation between the test (BOT-2 SF) and the criterion (BOT-2 CF). Thus, if we found that the correlation between the sum scores of the CF and SF was *r* = 0.76, it means that the relation between the SF and CF explains only 57% of variance in the BOT-2 CF. In addition to the correlation between the results of the CF and SF *r* = 0.76, which corresponded to the conclusions of previous studies, we also revealed a significant difference between the standard score achieved by the participants in the BOT-2 CF and BOT-2 SF and the results they achieved in the individual subtests. With the exception of strength and agility, lower correlations between the standard score and the score in the individual sub-test were always revealed in the BOT-2 SF, which is to be expected as the individual sub-tests in the BOT-2 SF include fewer indicators. In the BOT-2 SF, correlations between the standard score and the scores in fine manual control were significantly lower ES (Cohenq = 0.39 and body coordination Cohen *q* = 0.42). These results are in compliance with Brown's (22) findings who noted that nine out of 14 items in the BOT-2 SF do not meet the required level of fit. Five of these items belong to the sub-tests fine manual control and body coordination. A search of previous studies also showed that items of the following sub-tests—walking forward on a line, tapping feet, and fingers (same sides synchronized), jumping in place (same sides synchronized), drawing lines through paths (crooked)—repeatedly proved to be problematic (22–24).

To this it must be added that the strength of correlations in the BOT-2 is also influenced by the research sample, its compositions and specificity. For example, study (34) found a lower correlation between the CF and SF in a specific population group of football players. The correlation reached only *r* = 0.58. This value could be due to the low number of participants (*n* = 20, age 11.83 ± 0.25) and the specificity of the population (football players). Moreover, when we analyzed the data in our study with regard to the participants' sex, we found that the significance of differences in correlations between the scores in individual sub-tests and the standard scores of the BOT-2 CF and the BOT-2 SF is more noticeable in girls (Table 3). Therefore, we assume that the specificity and amount of participants have a large effect on the final value of correlation.

It must be pointed out that the BOT-2 SF is mainly used as a screening tool to achieve rapid and easy scoring reflecting the overall motor proficiency (8), which is also what Bruininks and Bruininks (12) claimed. Therefore, we were interested in how well the final interpretation of results from the BOT-SF will correspond with the final interpretation of results from the BOT-2 CF, in terms of whether the participants will achieve below-average, average or above-average results. Our results showed that BOT-2 SF has high sensitivity (84%) but low specificity (42.6%), which was also pointed out by McIntyre et al. (36). In our study, no participant with an above-average standard score in the BOT-2 CF achieved an above-average standard score in the BOT-2 SF. This finding means that the BOT-2 SF might be useful as a screening tool when screening children with possible delayed motor development but does not seem to be a suitable tool for identifying psychomotorically advanced children.

## Conclusion

From the practical point of view, the BOT-2 SF does not provide significantly different results from the BOT-2 CF when using proper scales for comparing both versions. In addition, the BOT-2 SF has acceptable sensitivity but poor specificity compared to the BOT-2 CF. Therefore, the BOT-2 SF might be a useful tool to reveal mainly delayed but not above-average (advanced) psychomotorically developed children. The relation between the SF and the CF explains only 57% of variance in the CF, which suggests that the CF and the SF do not measure exactly the same behavioral domain. Further research should focus on whether these differences are systematic or random. The BOT-2 SF can be used primarily as a field test for single-site screening mainly to identify children with suspicion for motor impairment.

## Ethics Statement

The procedures involved in our study were in accordance with the ethical standards of the responsible Czech national committee on human experimentation and with the Helsinki Declaration of 1975, as revised in 2000. The study was approved by the Ethics Commission of the Faculty of Physical Education and Sport, Charles University. Only children whose parents had signed the informed consent were recruited for the study.

## Author Contributions

JJ contributed to the introduction, data collection, data analysis, and discussion. MM contributed to the introduction, methods, discussion, and conclusion sections. FM contributed to the methods, discussion, and conclusion.

### Conflict of Interest Statement

The authors declare that the research was conducted in the absence of any commercial or financial relationships that could be construed as a potential conflict of interest.
